# Steroid Use, Adrenal Suppression, and Emergency Department Visits in COPD Patients: A Cross-Sectional Study

**DOI:** 10.2174/0118715303397988250428050120

**Published:** 2025-04-29

**Authors:** Talha Karahan, Nezihat Rana Dişel, Ömer Taşkın, Gülçin Dağlıoğlu, Mustafa Oğuz Tuğcan, Ahmet Sebe, Ayça Açıkalın Akpınar

**Affiliations:** 1 Department of Emergency Medicine, Kafkas University, Kars, Türkiye;; 2 Department of Emergency Medicine, Faculty of Medicine, Cukurova University, Adana, Türkiye;; 3 Department of Biochemistry, Faculty of Medicine, Cukurova University, Adana, Turkey;; 4 Department of Emergency Medicine, Adana Karşıyaka Hospital, Adana, Türkiye

**Keywords:** COPD exacerbation, steroids, adrenal suppression, cortisol, ACTH, systemic glucocorticoids

## Abstract

**Introduction:**

This study aims to investigate the relationship between steroid use, adrenal suppression, and frequent emergency department (ED) visits in patients with Chronic Obstructive Pulmonary Disease (COPD).

Systemic glucocorticoids are commonly prescribed in the management of COPD exacerbations; however, prolonged or repeated steroid use may lead to adrenal suppression. Although the standard steroid regimen for COPD exacerbations is short-term, frequent ED visits may result in cumulative steroid exposure, raising concerns about adrenal insufficiency and its clinical consequences.

This study investigates the potential association between steroid-induced adrenal suppression and frequent ED visits among COPD patients. It further examines the impact of steroid administration on cortisol and Adrenocorticotropic hormone (ACTH) levels.

**Methods:**

This prospective, cross-sectional, observational study was conducted in a university-based ED. Patients with COPD, with dyspnea and who presented to the ED between 06:00-08:00 were included. Demographics, previous presentations to the ED, medications used, hormone levels, and other laboratory results were recorded.

**Results:**

Fifty patients (82% were male) included. Sputum symptoms along with incidences of heart failure were higher in patients who received steroids in the ED. Ronchi was higher, crackles and pretibial edema were lower in the patients who received steroids in the ED. Among the patients with low cortisol levels, the frequency of patients who received steroids in the ED was higher than those who did not.

**Conclusion:**

Primary healthcare clinicians should monitor COPD patients for potential adrenal insufficiency. Careful regulation of steroid dosages during exacerbation treatment and minimizing polypharmacy are essential to mitigate the long-term effects of prolonged steroid use.

## 1 INTRODUCTION

 Chronic obstructive pulmonary disease (COPD) exacerbation occurs when there is a worsening of respiratory symptoms that requires additional and specific treatment [1-3]. Respiratory tract and lung infections are the most common cause of exacerbation in patients with COPD [4]. Several agents, including bronchodilators, corticosteroids, and anti-biotic combinations, are used in the treatment. The use of systemic glucocorticoids in the treatment of COPD exacerbation has been reported to shorten the recovery period, improve lung function, and increase oxygenation. On the other hand, it reduces the risk of early recurrence, treatment failure, and hospitalization [5-7]. Oral prednisolone is recommended at 40 mg daily for 5 days [8]. Its efficacy has been found to be equal to intravenous (IV) therapy [9].

 Corticosteroids, which are steroid hormones secreted from the adrenal cortex and analogous compounds with the same structure obtained by synthesis, are frequently used due to their anti-inflammatory, anti-allergic, and immunosuppressive effects [10]. Prolonged use of steroids can lead to adrenal suppression, which may impair the response to stress and complicate the fight against infection. The use of steroids in the treatment of COPD exacerbation does not align with the definition of “long-term steroid use” in the literature [11]. Patients with COPD usually receive steroid therapy for 5–10 days during hospital visits or when receiving outpatient care [8]. However, for patients with frequent emergency department (ED) presentations and hospitalizations, the duration and total dose may be higher.

 Our clinical experience indicates if a patient with COPD often presents to ED, physicians tend to administer steroids irrespective of whether the patient is experiencing an exacerbation, lung infection, or electrolyte deficiency as a side effect of bronchodilator medication. Patients tend to receive steroids at much higher doses than recommended in the guidelines. Although lung infections can trigger exacerbations, steroids can impair inflammatory responses, increasing susceptibility to and complicating all infections. This raises questions about whether steroids increase the frequency of exacerbations caused by lung infections in patients with COPD or whether they pave the way for sepsis originating from the lungs or elsewhere. Additionally, this raises the question of whether adrenal suppression occurs due to frequently given steroids, especially in patients with COPD who regularly visit EDs.

 This study seeks to determine whether steroid-induced adrenal suppression occurs in patients with COPD who commonly undergo repeated and high-dose steroid treatment and whether there is a relationship between steroid use and frequent ED presentations. In addition, the study aims to investigate the effects of steroid use in patients with COPD mortality.

## 2 MATERIALS AND METHODS

 This prospective, cross-sectional, observational study was conducted in a university-based ED after obtaining approval from the Çukurova University’s Non-interventional Clinical Research Ethics Committee prior to patient enrollment (approved in the committee meeting on June 1st, 2018 with ethical approval number #6). The study was supported by the Scientific Research Projects Fund of Çukurova University (ID number TTU-2018-10882).

 Patients with COPD, over 18 years of age, who were presented to the ED between May 1, 2018 and October 1, 2019, with dyspnea and gave written consent to participate were included. The patients were presented to the ED between 06:00-08:00 and whose blood sampling and adrenocorticotropic hormone (ACTH) and cortisol measurements could be taken before giving steroids in the ED. This timing was crucial because ACTH follows a diurnal rhythm, and a diagnosis of adrenal suppression could only be made by early morning sampling and steroid infusion could changed the cortisol levels.

 Patients were excluded from the study if they had known adrenal insufficiency or head trauma, who had undergone surgery and/or suffered hypovolemic hemorrhagic shock, who were referred to our tertiary center hospital for further treatment following a hospitalization period in another center, or whose ACTH and cortisol levels could not be assayed properly due to presentation earlier or later than optimal blood sampling time.

 The patients’ demographic data, complaints on presentation, comorbidities, medications, physical examination findings, number of presentations to the ED in the last six months, steroid use in the last three months, recent steroid use during the current ED visit, hospitalization status and duration, steroid use in the first month after presentation, one-month mortality, complete blood count, aspartate aminotransferase (AST), alanine aminotransferase (ALT), sodium, potassium, blood urea nitrogen (BUN), creatinine, lactate, c-reactive protein (CRP), procalcitonin (PCT), ACTH, cortisol levels were recorded. The patients’ data were evaluated according to whether steroids were given in the ED and whether they had used steroids in the previous three months.

### 2.1 Statistical Analysis

 The SPSS 23.0 package program was used for the statistical analysis of the data. Categorical measurements were reported as frequency and percentages, and continuous measurements were reported as mean±standard deviation and minimum–maximum levels. Pearson’s chi-square and Fischer’s exact test were used to compare categorical variables. For comparisons of continuous measurements between groups, Student’s t-test and Mann Whitney u test were used for binary variables by controlling the distributions. The statistical significance level was taken as 0.05 in all tests.

 Although a larger sample size was initially planned for this prospective study, the COVID-19 pandemic during the study period significantly impacted patient enrollment in the ED. Consequently, a post-hoc power analysis was performed to evaluate the adequacy of the achieved sample size (n=50). Based on the comparison of low cortisol rates between steroid-treated and non-treated patients, the statistical power was calculated as 76.3% (Cohen’s h=0.378, α=0.05), indicating sufficient power to detect moderate effects.

## 3 RESULTS

There were 50 patients, 41 (82.0%) of whom were male, who met the study’s inclusion criteria. The mean age of the patients was 66.72 years (range: 49–85 years). Thirty-seven (74%) patients were treated with steroids in the ED. There was no difference between the patients in terms of age and gender or whether they had taken steroids in the last three months or had received steroids in the ED. Cough was the most common complaint accompanying shortness of breath, occurring in 26 (52%) patients, and sputum occurred in 20 (40%) patients. One patient had impaired consciousness (2%) and one experienced respiratory arrest (2%). Hypertension (HT) (n=31, 62%) and coronary artery disease (CAD) (n=26, 52%) were the most common comorbidities. There was no statistically significant difference between the patients’ complaints except that sputum was found to be statistically higher (*p*=0.005) in the patients who received steroids in the ED. There was no statistically significant relationship between the patients’ comorbidities other than heart failure (HF), which was less common in the group who received steroids in the ED (*p*=0.007) (Table **1**).

There were crackles in 31 (62%), rhonchi in 43 (86%), pretibial edema in 17 (34%), jugular venous distension in 6 (12%) and hepatojugular reflux in 2 (4%) patients on physical examination at presentation. There was no statistically significant difference between the patients’ vitals except that the number of tachypneic patients who received steroids in the ED was statistically significantly higher than those who did not (*p*=0.049). There was no statistically significant difference between the patients’ findings of jugular venous distention (JVD) (*p*=0.173) and hepatojugular reflux (HJR) (*p*=0.456) whether they received steroids in the ED or not whilst crackles and rhonchi were found to be statistically significantly higher in those who received steroids in the ED, and pretibial edema (PTE) was found to be statistically significant in those who did not (*p*=0.049, *p*=0.000, *p*=0.003, respectively) (Table **2**).

 The mean number of ED visits in the last 6 months was 7.10 (range 0-30). Twenty-nine (58%) patients had used oral and/or inhaler steroids in the last 3 months, while 37 (74%) patients received steroids in the ED and 32 (64%) patients prescribed and used steroids within 1 month after presentation. The average number of hospitalization days was 10.04 (1-21 days) among the 30 (60%) hospitalized patients, 10 (20%) of which died within a month. There was no statistically significant difference between the status of the patients receiving steroids in the ED and the number ED visits in the last 6 months (p=0.667), oral and/or inhaled steroid use in the last 3 months (*p*=0.247) and in the first month following ED visit (*p*=0.542). Although the duration of hospitalization was shorter than those who received steroids in the ED, no statistically significant difference was found (*p*=0.465). It was determined that hospitalization status (*p*=0.582) and mortality in 1 month (*p*=0.485) were not different in terms of receiving steroid treatment in the ED (Table **3**).

 There was no statistical difference between the status of the patients receiving steroids in the ED and the laboratory results, except that high hemoglobin (Hb) (*p*=0.011) and Hematocrit (Hct) levels (*p*=0.018) were found in the group who received steroids in the ED (Table **4**).

 There was no difference between the ACTH levels of the patients, but the frequency of the patients with low levels of cortisol was higher in the patients who received steroids in the ED (*p*=0.009) (Table **5**).

## 4 DISCUSSION

 This study examined the effect of steroid therapy, which is frequently used in EDs in COPD patients, on the hormones of the adrenal gland and its effects on the ED visits of patients. The findings of the study indicated that steroid use-induced adrenal suppression may occur in COPD patients. In this section, the findings will be discussed with the current literature.

 Besides the need for long-term hospitalization, patients having COPD with high mortality and morbidity may also be discharged even from the ED following treatment of an exacerbation [12-15]. The incidence of COPD in Türkiye is between 15% and 20% in adults over the age of 40. Hacıevliyagil *et al.* reported 61.9 years and Varol *et al.* reported 65.3 years of age for the COPD population in Türkiye in their studies [13, 15]. Shingol *et al.* [16] reported that COPD is more common in males, with a rate of 77.8% and Kalvaci *et al.* [17] reported a rate of 91.3%. In this study, male patients were approximately 4 times more than females and the ages of patients were similar to the ages of COPD patients in the Turkish population.

 In patients with COPD, dyspnea is the first and most common symptom, the severity of which increases gradually, affects the general condition, and causes early mortality [16]. Cough and sputum adversely affect the quality of life of patients with COPD and cause more medical treatments, hospitalizations, and premature death [17]. In this study, dyspnea was the inclusion criterion, and other existing symptoms were found to be compatible with the literature. In our study, the group of patients who received steroids presented with a lower frequency of crackles and orthopnea but a higher frequency of rhonchi and sputum. Additionally, the proportion of patients with elevated respiratory rates was greater in the steroid-treated group. These findings suggest that physicians might have considered the presence of COPD exacerbation–like symptoms (*i.e.*, increased dyspnea due to rhonchi and sputum) as a clinical cue for administering steroids. This may have introduced a degree of clinical bias in treatment decisions based on presenting respiratory symptoms.

 In a study of patients with COPD who died during hospitalization, it was reported that cardiovascular diseases and diabetes mellitus (DM) were the most common comorbidities with COPD [18]. Yıldırım *et al.* reported at least one comorbidity in 80.8% of their study patients [19]. Cor pulmonale, respiratory failure and HT were found to be more common in a study by Chen *et al.* [20]. HT, CAD and HF were the most common comorbidities in this study, and HF was statistically significantly lower in patients who received steroids in the ED. It is known that steroids are not recommended by the guidelines of HF, for both children and adults [21, 22]. It can be said that the decision of using steroids in the treatment of acute exacerbations is affected by the patients having HF or not as more patients without HF received steroids in the ED. We believe that HF is a confounding factor influencing the decision to administer steroids.

 In COPD, the relationship between the clinical presentation and the severity of the airway obstruction is weak, and the physical examination and vital parameters of patients who appear clinically very poor may be within normal limits [23]. It is known that the basal respiratory rate of patients with COPD is high and they are hypercarbic, hypoxemic and have low oxygen saturations. In this study, while tachycardia was observed in approximately half of the patients applied to the ED, respiratory rate was high in almost all. In this study, patients who received steroids in the ED, crackles, rhonchi, and pretibial edema were found to be statistically higher than those who did not. Patients with rhonchi seem to be evaluated as they needed steroids by the attending physician. In previous studies conducted in the ED, the presence of rhonchi and crackles in patients requiring hospitalization and the rate of crackles, rhonchi and pretibial edema in cancer patients were found to be high [24].

 Ely *et al.* [25] reported that the duration of hospitalization for COPD was longer and significantly higher. In a study conducted in the ED, 62.8% of the patients who presented with acute COPD exacerbations were discharged from the ED, 34.4% were hospitalized, 2.1% refused treatment and left, and 0.7% deceased in the ED [26]. The hospitalization ratio of the patients in this study was consistent with the literature; there was no mortality in the ED, but 20% of the patients deceased within the first month.

 Oral steroids are found to shorten the length of hospitalization in severe exacerbations [27]. Patients in this study who received steroids in the ED spent an average of 9.68 days in hospital, which was lower than those who did not receive steroids. The number of ED visits in the last 6 months was higher in the patients who received steroids in the ED. Most of the patients (23/37) who received steroids in the ED had used oral and/or inhaled steroids in the last 3 months, and the number of patients who continued using steroids in the month following discharge was also higher (24/37) than those who did not receive steroids. These results were not statistically significant due to the small number of our sample size, but these findings are engrossing.

 Studies have shown that CRP is elevated in acute exacerbations of COPD. CRP levels and platelet count of patients with COPD are higher than the healthy control group without airway restriction and reported to be related to increased risk of acute exacerbations [28-30]. In this study, the Hb and Hct values were higher in those who received steroids in the ED. Lactate, CRP, ALT, creatinine, BUN levels and platelet count in patients who received steroids in the ED were higher than those who did not, but not statistically significant, which may be due to the sample size.

 While 18 of the patients had low cortisol levels and 15 of these were given steroids in the ED, there were 32 patients with normal and above normal cortisol levels. These results suggest that there may be adrenal suppression. Furthermore, the response to stress may be negatively affected in these patients, their clinical course may be poor in the current attack of COPD, and they constitute the patient group that requires steroids in addition to bronchodilators in the management of acute exacerbations. The cortisol levels of these patients were low when they were under severe stress during the hypoxic phase of the attack. On the other hand, although there is no statistical significance, the fact that ACTH was below the normal value in 13 patients, 9 of whom were given steroids in the ED, deserves to be investigated for tertiary adrenal insufficiency. It is known that high cortisol affects ACTH secretion with negative feedback [31]. An increase or decrease in serum cortisol level causes a change in ACTH levels in the opposite direction. While external glucocorticoid intake suppresses ACTH secretion (tertiary adrenal insufficiency), ACTH level increases in individuals who have undergone adrenalectomy [31]. Although the recommended steroid dose for patients with COPD is 40 mg/day for 5-7 days, we observed that the frequent emergency visits and repetitive exposure to IV/oral/inhaler steroids caused higher total doses than recommended in the GOLD guidelines [9]. We expect ACTH and cortisol levels to be low in steroid users. Although the steroid dose received by patients with COPD is lower than in chronic high-dose corticosteroid treatments, drugs that affect cortisol metabolism, such as CYP3A4 inhibitors, may cause prolonged action and, thus, adrenal suppression. An example of this in the literature is the antiviral ritonavir [13]. However, clarithromycin is also a CYP3A4 inhibitor and is an antibiotic that is frequently prescribed for outpatient treatment of COPD pneumonia caused by atypical agents.

Exogenous steroid intake will first suppress ACTH secretion, but then the decreased endogenous cortisol synthesis of the patient, who is freed from exogenous steroids over time, may cause elevated ACTH levels. While the ACTH levels of 32 of our patients were within the normal range, higher values were found in 5 patients. Here, the ACTH levels of the patients should only be evaluated on a patient-by-patient basis according to their cortisol levels, which is out of the scope of this study. However, this is an important issue to study in this patient group with chronic steroid use (although it does not fit the description of the classical high dose). Among the patients with low cortisol levels, the frequency of patients who received steroids in the ED was higher than those who did not. Patient's complaints and comorbities

 Could the repeated use of steroids, both before and during treatment, have caused low cortisol levels? These patients are known for frequent ED admissions and hospitalization. We speculate patients with COPD might experience central or adrenal suppression due to chronic hypoxic hypercarbia processes. The patient with known HF seems to be protected from extra steroid doses to keep their volume status in balance, thus, this might affect the treatment choice of the physicians for steroid free treatment strategies. Moreover, we believe that the patients who received steroids in the ED might have opted because of suppressed pituitary and adrenals, resulting in low endogenous cortisol levels and inappropriate response to stress. Lastly, the patients who receive steroids during COPD exacerbations in each ED visit are meant to receive some hormone replacement therapy due to their ongoing deficiencies.

## 5 LIMITATIONS

 The most important limitation of our study is the small sample size due to the optimum ACTH and cortisol sampling time. Moreover, we do not know the previous cumulative steroid amounts used due to the low reliability of anamnesis since the exact amount of steroids taken by the patients at other hospital admissions and each application could not be tracked. The decision not to administer steroids for controlling COPD exacerbations may have been influenced by patients’ past medical histories and physical examination findings. The lack of a standardized treatment protocol among physicians could have contributed to variability in clinical decisions, particularly leading to lower steroid use in patients with heart failure.

## CONCLUSION

We believe that it would be beneficial to follow up patients with COPD in terms of adrenal insufficiency by primary healthcare clinicians. It is crucial to control the amount of steroids administered during the treatment of exacerbations and to reduce polypharmacy to reduce prolonged steroid effects, to examine the previous steroid use histories of the patients presenting with exacerbation and infection, and to examine the patients in terms of adrenal insufficiency. Adrenal insufficiency should be kept in mind as one of the causes of frequent ED visits in patients with COPD.

## Figures and Tables

**Table 1:** Patient's complaints and comorbities.

-		**Total**	**Treated with steroids in the ED**		***p***
			**Non-receivers**	**Receivers**	
Patient’s complaints, n (%)	Shortness of breath	50 (100)	13 (100)	37 (100)	1.00
	Loss of consciousness	1 (2)	1 (7.7)	0 (0)	0.26
	Palpitations	7 (14)	2 (15.4)	5 (13.5)	0.594
	Pre/syncope	3 (6)	1 (7.7)	2 (5.4)	0.604
	Cough	26 (52)	5 (38.5)	21 (56.8)	0.208
	Sputum	20 (40)	1 (7.7)	19 (51.4)	**0.005**
	Fever	12 (24)	1 (7.7)	11 (29.7)	0.107
	Chest pain	8 (16)	2 (15.4)	6 (16.2)	0.659
Patient’s comorbidities, n (%)	Diabetes mellitus	14 (28)	5 (38.5)	9 (24.3)	0.264
	Coronary artery disease	26 (52)	0 (69.2)	17 (45.9)	0.131
	Hypertension	31 (62)	9 (69.2)	22 (59.5)	0.39
	Heart failure	22 (44)	10 (76.5)	12 (32.4)	**0.007**
	Chronic liver disease	2 (4)	0 (0)	2 (5.4)	0.544
	Cerebrovascular disease	4 (8)	0 (0)	4 (10.8)	0.287
	Renal failure	2 (4)	0 (0)	2 (5.4)	0.544
	Lung cancer	3 (6)	0 (0)	3 (8.1)	0.396
	Other malignities	4 (8)	2 (15.4)	2 (5.4)	0.275

**Table 2:** Patient's vitals and findings at presentation.

-			**Total**	**Treated with steroids in the ED**		***p***
				**Non-receivers**	**Receivers**	
Vital’s on presentation, n (%)	Body temperature (°C), *mean±SD (min-max)*		36.74±0.86 (35.5-39.6)	36.85±1.01 (35.8-39.6)	36.70±0.82 (35.5-39.1)	0.608
	Systolic BP	Low (≤ 89 mmHg)	0 (0)	0 (0)	0 (0)	0.514
		Normal (90-139 mmHg)	29 (58.0)	8 (61.5)	21 (56.8)	
		High (≥140 mmHg)	21 (42.0)	5 (38.5)	16 (43.2)	
	Diastolic BP	Low (≤59 mmHg)	5 (10.0)	1 (7.7)	4 (10.8)	0.793
		Normal (60-89 mmHg)	39 (78.0)	11 (84.6)	28 (75.7)	
		High (≥90 mmHg)	6 (12.0)	1 (7.7)	5 (13.5)	
	MAP	Low (≤69 mmHg)	1 (2.0)	1 (7.7)	0 (0.0)	0.160
		Normal (70-100 mmHg)	36 (72.0)	10 (76.9)	26 (70.3)	
		High (≥101 mmHg)	13 (26.0)	2 (15.4)	11 (29.7)	
	Pulse	Normal (60-100 bpm)	26 (52.0)	8 (61.5)	18 (48.6)	0.318
		High (≥101 bpm)	24 (48.0)	5 (38.5)	19 (51.4)	
	Respiratory rate	Normal (12-20 bpm)	4 (8.0)	3 (23.1)	1 (2.7)	**0.049**
		High (≥20 bpm)	46 (92.0)	10 (76.9)	36 (97.3)	
	Oxygen Saturation	<90%	23 (46.0)	4 (30.8)	19 (51.4)	0.170
		≥90%	27 (54.0)	9 (69.2)	18 (48.6)	
Findings at presentation, n (%)	Crackles		31 (62)	11 (84.6)	20 (54.1)	**0.049**
	Ronchi		43 (86)	6 (46.2)	37 (100)	**<0.001**
	Pretibial edema		17 (34)	9 (69.2)	8 (21.6)	**0.003**
	Jugular venous distension		6 (12)	3 (23.1)	3 (8.1)	0.173
	Hepatojugular reflux		2 (4)	1 (7.7)	1 (2.7)	0.456

**Abbreviations:** SD, standard deviation; BP, blood pressure; MAP, mean arterial pressure; ED, emergency department.

**Table 3:** Emergency department visits, hospitalization, steroid use and mortality in one month of patients.

-		**Total**	**Treated with steroids in the ED**		***p***
			**Non-receivers**	**Receivers**	
Number of ED visits in the last 6 months, mean±SD (min-max)		7.1±7.59 (0-30)	6.31±6.70 (0-20)	7.38±7.95 (0-30)	0.667
Duration of hospitalization (days), *mean±SD (min-max)*		10.04±5.17 (1-21)	11.6±5.22 (7-20)	9.68±5.22 (1-21)	0.465
Oral and/or inhaled steroid use in the last 3 months, n (%)	No	21 (42)	7 (53.8)	14 (37.8)	0.247
	Yes	29 (58)	6 (46.2)	23 (62.2)	
Steroid use in the first month following ED visit, n (%)	No	13 (26)	5 (38.5)	13 (35.1)	0.542
	Yes	37 (74)	8 (61.5)	24 (64.9)	
Hospitalization status, n (%)	No	20 (40)	5 (38.5)	15 (40.5)	0.582
	Yes	30 (60)	8 (61.5)	22 (59.5)	
Mortality in one month, n (%)	No	40 (80)	11 (84.6)	29 (78.4)	0.485
	Yes	10 (20)	2 (15.4)	8 (21.6)	

**Abbreviations:** ED, emergency department; SD, standard deviation.

**Table 4:** Laboratory results of the patients.

-	**Total**	**Treated with steroids in the ED**		***p***
		**Non-receivers**	**Receivers**	
Lactate (125-225 U/L), mean±SD (min-max)	2.82±1.73 (0.93-12.57)	2.57±1.09 (0.93-4.09)	2.90±1.91 (1-12.57)	0.551
PCT (0.12-0.36 ng/ml), *mean±SD (min-max)*	0.23±0.48 (0.0-2.44)	0.26±0.66 (0.02-2.44)	0.22±0.41 (0.0-2.25)	0.769
CRP (0-5 mg/L), *mean±SD (min-max)*	38.07±53.66 (0.5-311)	21.58±19.58 (1.97-55.90)	43.87±60.49 (0.5-311.0)	0.201
AST (15-41 U/L), *mean±SD (min-max)*	30.48±21.90 (9-112)	36.85±28.65 (13-112)	28.24±18.96 (9-105)	0.227
ALT (7-35 U/L), *mean±SD (min-max)*	19.98±10.32 (4-56)	17.38±8.10 (4-30)	20.89±10.94 (7-56)	0.297
Sodium (136-146 mmol/L), *mean±SD (min-max)*	137.1±4.27 (125-143)	135.38±6.29 (125-143)	137.78±3.19 (133-143)	0.082
Potassium (3.6-5.1 mmol/L), *mean±SD (min-max)*	4.31±0.56 (3.2-5.5)	4.10±0.68 (3.2-5.3)	4.38±0.50 (3.6-5.5)	0.121
Creatinine (0.4-1 mg/dl), *mean±SD (min-max)*	0.84±0.33 (0.32-2.26)	0.69±0.26 (0.32-1.08)	0.89±0.34 (0.43-2.26)	0.062
BUN (8-20 mg/dl), *mean±SD (min-max)*	21.37±8.46 (8.14-44.57)	19.08±9.46 (8.72-44.3)	22.17±8.07 (8.14-44.57)	0.261
WBC (4.8-10.8x10^3^/μL), *mean±SD (min-max)*	14.38±10.93 (2.7-68.3)	14.71±14.91 (4.9-62.9)	14.38±10.93 (2.7-68.3)	0.933
Platelet (130-400 x10^3^/mm^3^), *mean±SD (min-max)*	250.14±120.75 (22-603)	266.07±153.38 (87-603)	244.54±109.0 (22-502)	0.585
Hb (12-16 g/dL), *mean±SD (min-max)*	12.95±2.20 (7.8-20.0)	11.63±1.59 (9-13.9)	13.41±2.22 (7.8-20.0)	**0.011**
HCT (35-50 (%)), *mean±SD (min-max)*	39.16±6.22 (23.1-60.0)	35.70±4.23 (28.1-42.1)	40.37±6.40 (23.1-60.0)	**0.018**

**Abbreviations:** ACTH, adrenocorticotropic hormone; AST, aspartate aminotransferase; ALT, alanine aminotransferase; BUN, blood urea nitrogen; CRP, c-reactive protein; PCT, Procalcitonin; ED, emergency department; SD: standard deviation.

**Table 5:** ACTH and cortisol levels of the patients.

-		**Total**	**Treated with steroids in the ED**		***p***
			**Non-receivers**	**Receivers**	
ACTH (10-50 pg/ml), n (%)	Low	13 (26.0)	4 (30.8)	9 (24.3)	0.623
	Normal	32 (64.0)	7 (53.8)	25 (67.6)	
	High	5 (10.0)	2 (15.4)	3 (8.1)	
Cortisol (6.7-22.6 µg/dl), n (%)	Low	18 (36.0)	3 (23.1)	15 (40.5)	**0.009**
	Normal	29 (58.0)	7 (53.8)	22 (59.5)	
	High	3 (6.0)	3 (23.1)	0 (0.0)	

**Abbreviations:** ACTH, Adrenocorticotropic hormone; ED, emergency department.

## Data Availability

The data and supportive information are available within the article.

## STANDARDS OF REPORTING

STROBE guidelines were followed.
